# Design of a TDOA location engine and development of a location system based on chirp spread spectrum

**DOI:** 10.1186/s40064-016-3632-0

**Published:** 2016-11-14

**Authors:** Rui-Rong Wang, Xiao-Qing Yu, Shu-Wang Zheng, Yang Ye

**Affiliations:** 1 Department of Instrumentation Science and Technology, Hangzhou Dianzi University, BaiYang Street, Hangzhou, 310018 China; 2 Department of Health Management and Hospital Management, Hangzhou Red Cross Hospital, Around the City East Road, Hangzhou, 310003 China; 3 Department of Detection Technology and Automation Equipment, Hangzhou Dianzi University, BaiYang Street, Hangzhou, 310018 China

**Keywords:** Chirp spread spectrum (CSS), Time difference of arrival (TDOA), Kalman, Taylor, Wireless sensor networks (WSN)

## Abstract

Location based services (LBS) provided by wireless sensor networks have garnered a great deal of attention from researchers and developers in recent years. Chirp spread spectrum (CSS) signaling formatting with time difference of arrival (TDOA) ranging technology is an effective LBS technique in regards to positioning accuracy, cost, and power consumption. The design and implementation of the location engine and location management based on TDOA location algorithms were the focus of this study; as the core of the system, the location engine was designed as a series of location algorithms and smoothing algorithms. To enhance the location accuracy, a Kalman filter algorithm and moving weighted average technique were respectively applied to smooth the TDOA range measurements and location results, which are calculated by the cooperation of a Kalman TDOA algorithm and a Taylor TDOA algorithm. The location management server, the information center of the system, was designed with Data Server and Mclient. To evaluate the performance of the location algorithms and the stability of the system software, we used a Nanotron nanoLOC Development Kit 3.0 to conduct indoor and outdoor location experiments. The results indicated that the location system runs stably with high accuracy at absolute error below 0.6 m.

**Electronic supplementary material:**

The online version of this article (doi:10.1186/s40064-016-3632-0) contains supplementary material, which is available to authorized users.

## Background

In recent years, wireless localization technology has become very popular in industrial, commercial, military, and other fields as an effective approach to estimating the characteristic parameters of a given signal, as well as obtaining the location information of target location nodes (Tag) through a variety of physical measurements (Liu et al. 2007; Lim et al. 2007; Golden and Bateman 2007; Wang et al. 2013). Notable examples include global positioning systems (GPS), WiFi, ZigBee, ultrasound, ultra-wide bands (UWB), and CSS. The most recent research (Rabinowitz and Spilker 2005; Gozick et al. 2011) has been focused on broadcast signals and geomagnetism.

Although the GPS and cellular location services are common, it is still challenging to deploy them in certain complex application environments, especially indoor environments. The accuracy of cellular network locations decreases significantly under non-line of sight (NLOS) signal propagation and multi-path interference. New wireless network technologies and corresponding positioning methods such as ZigBee (Blasco et al. 2009), Bluetooth (Jan et al. 2014), radio frequency identification (RFID) (Zhou and Shi 2009), UWB and CSS have become attractive potential solutions to this problem. Positioning systems based on RFID, Wi-Fi (Figuera et al. 2011) and ZigBee have lower precision than UWB and CSS, as the latter are able to restrain NLOS propagation error, consume relatively little power, and have high ranging precision. Unfortunately, the physical characteristics of UWB propagation tend to interfere with other narrow band wireless communication systems (Hamalainen et al. 2002). UWB equipment also costs much more than other, similar equipment. Though CSS equipment may have 1–2 m error (Nanotron Technologies GmbH 2005), it still can meet the requirements of most location areas; combined with its advantages of lower cost and less interference compared to other wireless systems, CSS is the best choice for many areas.

**Fig. 1:**
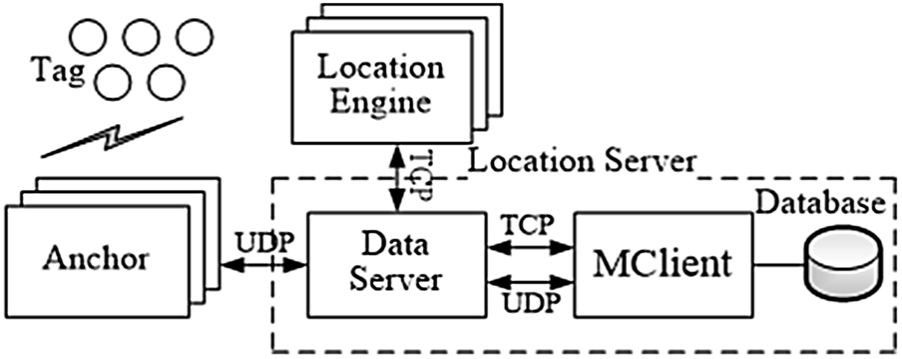
Software framework of proposed location system

**Fig. 2:**
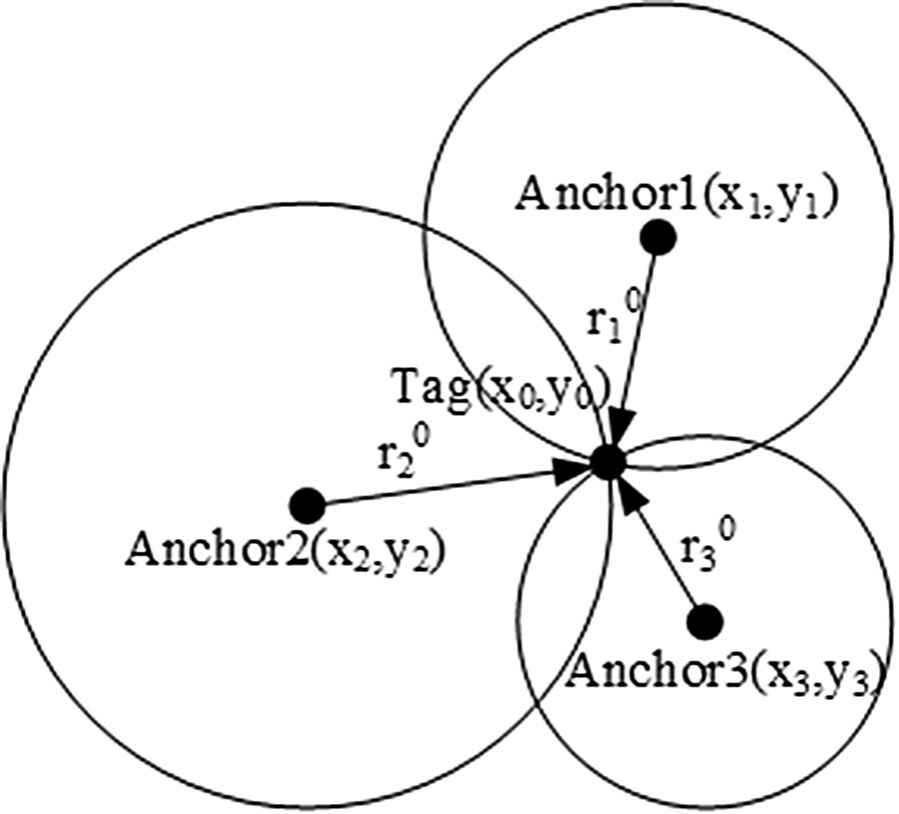
TOA localization model

**Fig. 3:**
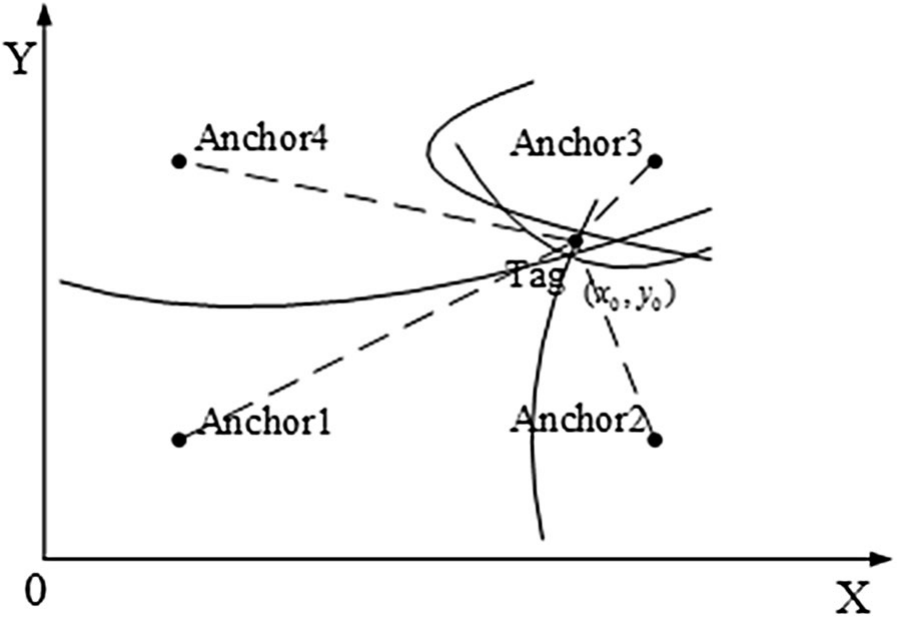
TDOA localization model

**Fig. 4:**
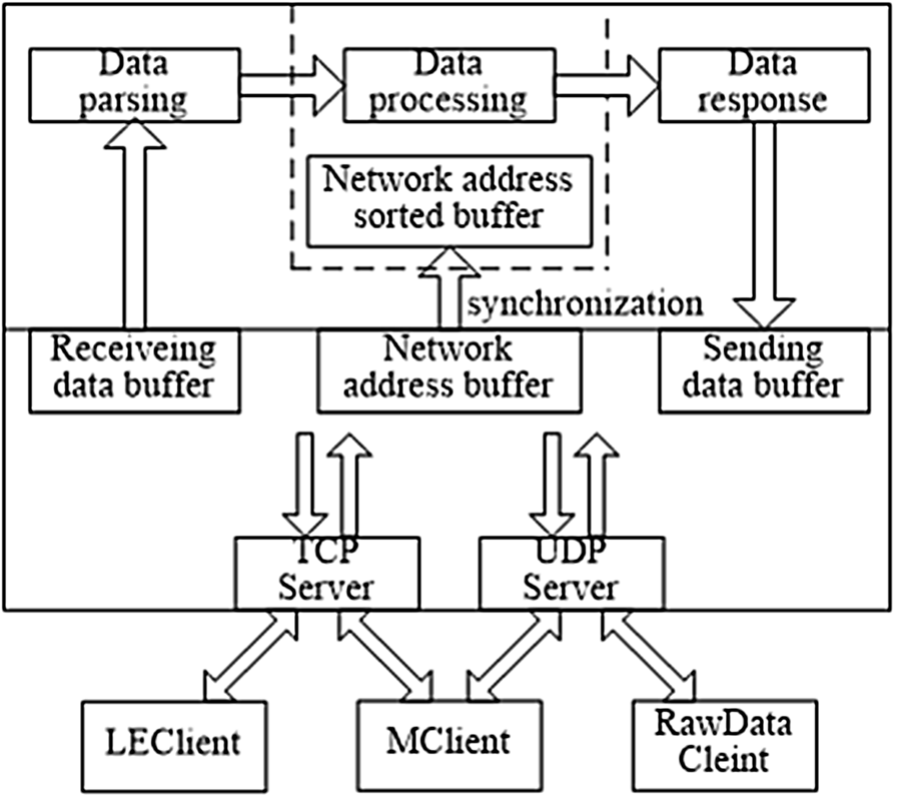
Data server framework

**Fig. 5:**
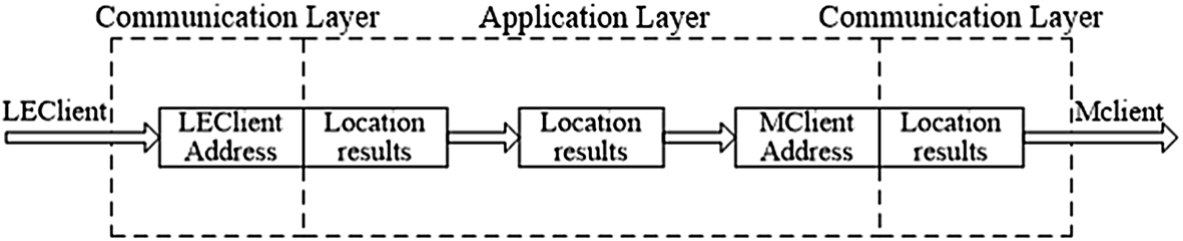
Flow chart of location results in data server

**Table 1:** Comparison of mean values of location results in the indoor environment (unit: m)

Reference	Chan	Taylor	Kalman	Compared Taylor	This paper
(2.26, 1.80)	(2.93, 2.80)	(2.30, 2.20)	(2.26, 2.21)	(2.30, 2.20)	(2.28, 2.21)
(2.26, 3.60)	(2.82, 3.24)	(2.62, 4.02)	(2.76, 3.95)	(2.64, 4.00)	(2.68, 3.98)
(3.39, 0.00)	(3.08, 0.74)	(3.43, 0.03)	(3.38, 0.03)	(3.43. 0.03)	(3.41, 0.00)
(3.39, 1.80)	(3.25, 2.35)	(3.83, 1.59)	(3.99, 1.63)	(3.85, 1.65)	(3.85, 1.71)
(3.39, 3.60)	(3.23, 2.96)	(3.74, 3.42)	(3.65, 3.32)	(3.74, 3.42)	(3.70, 3.36)
(3.39, 5.40)	(3.11, 3.91)	(3.60, 5.16)	(3.52, 4.92)	(3.52, 5.16)	(3.50, 5.18)

**Table 2:** Comparison of RMSE values of location results in the indoor environment (unit: m)

Reference	Chan	Taylor	Kalman	Compared Taylor	This paper
(2.26, 1.80)	1.47	0.44	0.42	0.44	0.42
(2.26, 3.60)	0.76	0.64	0.64	0.67	0.57
(3.39, 0.00)	0.91	0.15	0.09	0.15	0.09
(3.39, 1.80)	1.15	0.74	0.76	0.64	0.60
(3.39, 3.60)	0.66	0.40	0.39	0.40	0.38
(3.39, 5.40)	2.13	0.87	0.77	0.55	0.40

All available localization methods can be divided into two categories: Range-based or range-free (Figuera et al. 2011), based on whether the distance must be ranged or not. Range-free location systems first establish a fingerprint database by collecting the received signal strength indication (RSSI) data between the Tags and Anchors to obtain a signal propagation model, then real-time RSSI parameters are measured and matched to the fingerprint database to estimate the coordinate value of Tag. Range-based localization methods (Dan 2011; Guvenc and Chong 2009; Ho 2012), conversely, include angle of arrival (AOA), RSSI, time of arrival (TOA), and TDOA.

**Fig. 6:**
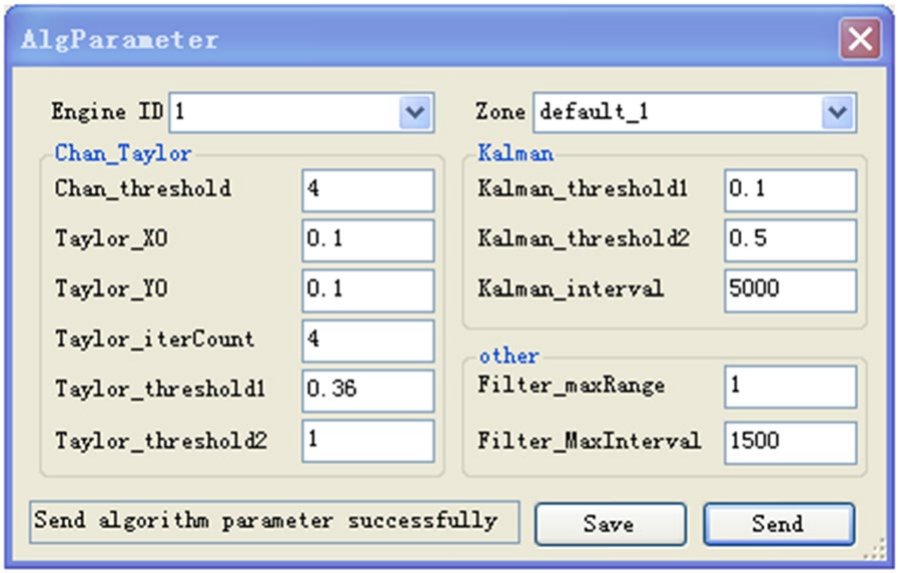
Set algorithm parameter of location system

**Fig. 7:**
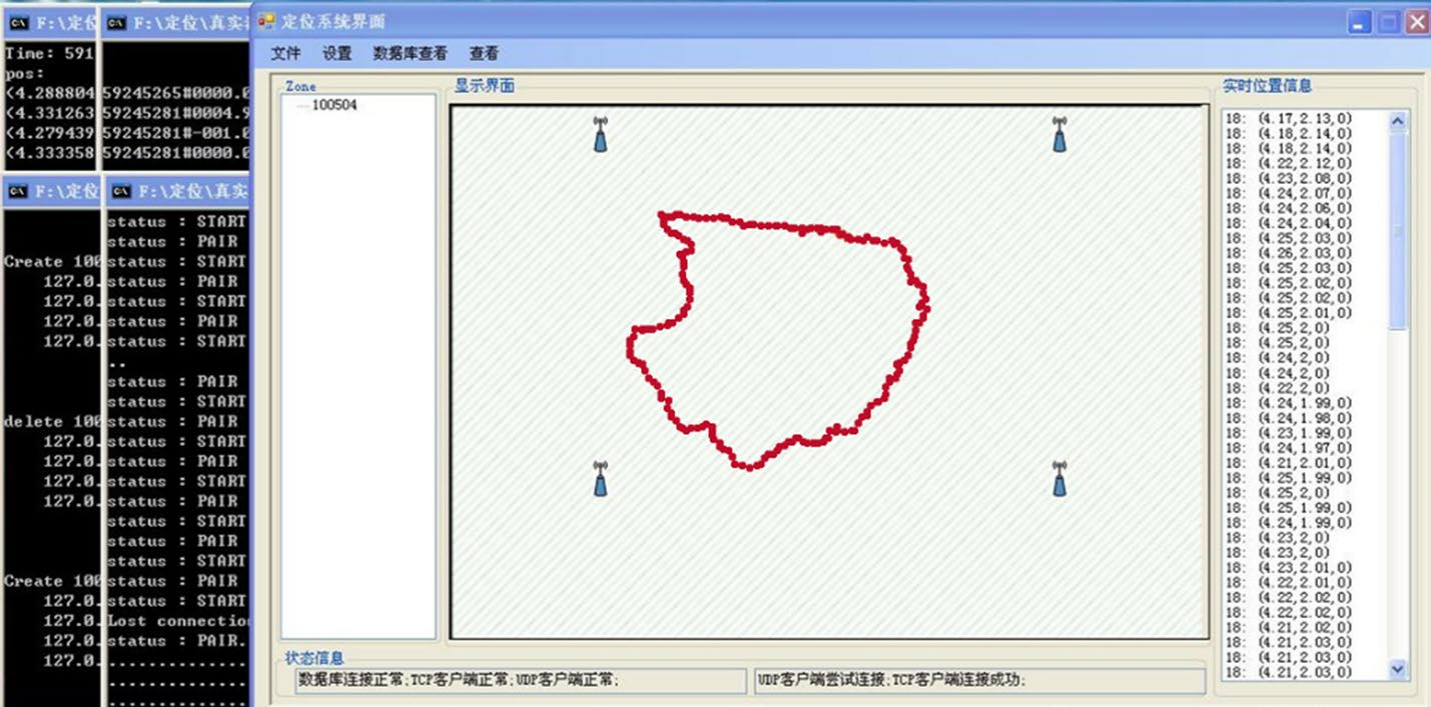
Display interface of simulation location system

**Fig. 8:**
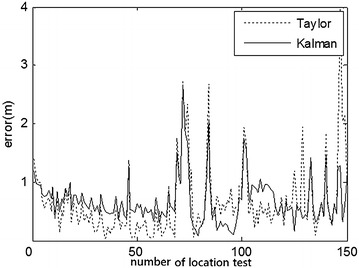
Comparison of indoor position errors of Taylor and Kalman methods (3.39, 5.40)

**Fig. 9:**
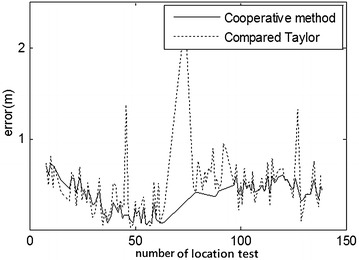
Comparison of indoor position errors Taylor and cooperative method (3.39, 5.40)

**Fig. 10:**
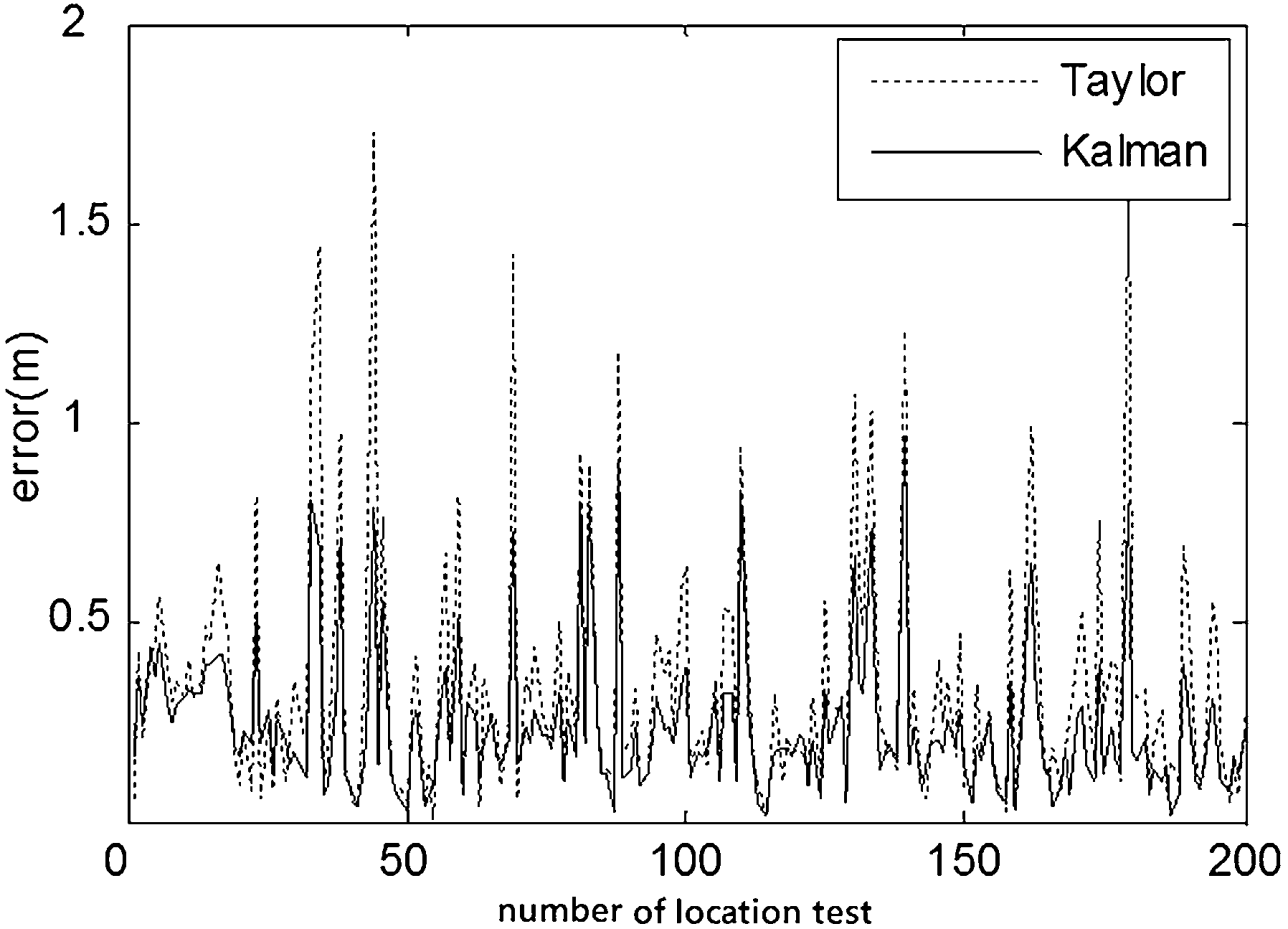
Comparison of outdoor position errors of Taylor and Kalman methods (3.00, 4.00)

**Fig. 11:**
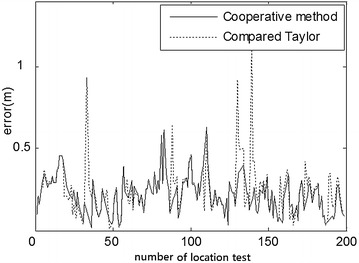
Comparison of outdoor position errors Taylor and cooperative method (3.00, 4.00)

**Fig. 12:**
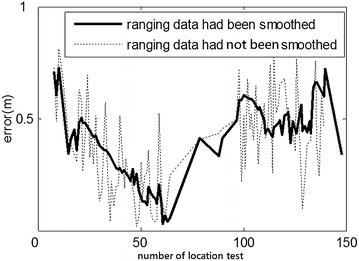
Comparison of error for smoothed/unsmoothed ranging data in the indoor position (3.39, 5.40)

**Fig. 13:**
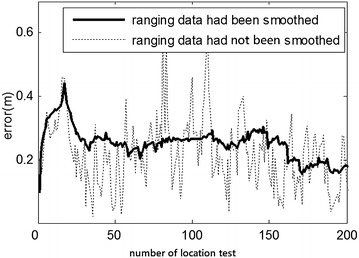
Comparison of the error for smoothed/unsmoothed ranging data in the outdoor position (3.00, 4.00)

**Table 3:** Comparison of mean values of location results in the outdoor environment (unit: m)

Reference	Chan	Taylor	Kalman	Compared Taylor	This paper
(0.00, 4.00)	(1.72, 3.98)	(0.07, 4.04)	(−0.02, 4.00)	(0.12, 4.04)	(0.17, 4.05)
(3.00, 4.00)	(2.80, 4.01)	(3.24, 4.00)	(3.24, 4.00)	(3.22, 4.00)	(3.23, 4.01)
(5.00, 4.00)	(2.98, 4.03)	(5.42, 4.07)	(5.54, 4.07)	(5.40, 4.07)	(5.48. 4.02)

**Table 4:** Comparison of RMSE values of location results in the outdoor environment (unit: m)

Reference	Chan	Taylor	Kalman	Compared Taylor	This paper
(0.00, 4.00)	1.92	0.50	0.32	0.39	0.22
(3.00, 4.00)	0.34	0.43	0.34	0.39	0.27
(5.00, 4.00)	2.34	0.73	0.65	0.60	0.51

The TDOA model can be used to measure the differences in the times at which signal from the Tag directly or indirectly arrive at multiple Anchors. Because the TDOA model only requires clock synchronization between the Anchors, its hardware equipment can be more simply and easily implemented than the TOA model. The TDOA model is also lower in cost than the AOA, and has stronger anti-interference ability than RSSI (as the signal information of RSSI is vulnerable to factors like temperature, space, scene, or change in receiving terminals Yousef et al. 2003; Sayed et al. 2005).

Chan, Taylor, extended Kalman filter (EKF), and particle filter (PF), et al., are frequently-used algorithms in TDOA location. The positioning precision of Chan algorithm decreases significantly in NLOS environment. Taylor algorithm can obtain accurate computation when initial estimated situation approximates the actual location, otherwise, it is difficult for the algorithms to ensure the convergence. But PF algorithm performs on poor instantaneity. The proposed algorithm has high accuracy and performs well in real-time tracking. A novel, CSS-based location system including a TDOA ranging method is presented in this paper. A location engine was designed as a series of location algorithms and smoothing algorithms, and a Kalman filter algorithm and moving weighted average technique were respectively applied to smooth the TDOA range measurements and location results.

## Methods

### Concept design of wireless location system

#### Wireless location system framework

The positioning system is primarily divided into three parts: The nodes (Anchor and Tag), the location server, and the location engine. The location nodes in our system were designed with a ranging module based on the CSS technology produced by Nanotron Company; the location server collects the TDOA measurements and transmits them to the location engine. The server must effectively manage the configurations of Tags, Anchors, and location engines, such as by specifying the parameters of the location algorithms, initializing the coordinate values of the Anchors, and selecting the necessary algorithm. To simplify the system, our location server was divided into two parts: MClient (interface management client) and Data Server. The Data Server was designed as an information processing center, while the MClient was designed as an information management center able to access the database directly.

The location engine receives the measurements and generates the ultimate location results, as well as the coordinate values of the Tags with the location algorithms in real-time. Detailed TDOA location algorithms provided by Chan et al. (2006), Foy (1976) and Li and Liu (2005), and data smoothing methods were coded into the location engine using programming language C++. The location results are sent to the server again and stored in a database, then location results are displayed in real-time on the Mclient in graphic form. The main software framework is shown in Fig. 1.

### Design and implement of location engine

The communication network protocol between the location engine and the data server is TCP. The necessary algorithms were derived according to the Anchor coordinates and algorithm parameters, then TDOA measurements were input into the formulas to produce location result outcomes. The Location Engine is tasked with sending these results to the server. To improve the computational efficiency, the Location Engine caches additional data (Anchor coordinates and algorithm parameters). A specific calculation process including a TDOA measurement smoothing method based on Kalman Filtering, a cooperative localization method based on Kalman algorithm and Taylor algorithm was built to ensure precise and real-time results.

### TDOA positioning principle

Again, the TDOA model measures the differences in times at which signals from the Tag directly or indirectly arrive at multiple Anchors; the TOA model only measures the time at which the signal arrives at one Anchor from the Tag. Anchors are devices placed at fixed sites with known coordinate values, while the coordinates of Tag devices must be estimated according to the anchor values and measurements. The measurement accuracy of TDOA depends on two factors: Accurate recording of the arriving time, and clock synchronization accuracy between the various Anchors. Measurements are then used to establish a hyperbolic model and to estimate the Tag coordinates. The hyperbolic model of TDOA is more complex than the circle model of TOA, accordingly, as depicted in Figs. 2 and 3. TOA obtains measurements as the signal propagation time between Tag and the *i*th Anchor, then the distance *r*th between the Tag and the *i*th Anchor can be calculated as follows:

1 $$\begin{aligned} r_i=t_i*v \quad i=1,2,3,\ldots ,n \end{aligned}$$

where *v* represents the speed of light. TDOA returns the measurement $t_{ij}$, i.e., the time difference of the signals arrival at the *i*th Anchor and *j*th Anchor from the Tag. The distance difference $r_{ij}$ can be obtained as follows:

2 $$\begin{aligned} r_i-r_j=r_{ij}=t_{ij}*v \quad i=1,2,3,\ldots ,n \end{aligned}$$

Figures 2 and 3 show where the TOA model obtains the Tags location by finding $n(n\ge 3)$ circles intersections, then the TDOA model obtains the $n(n\ge 4)$ hyperbolas intersection. There is no sizable difference between TOA and TDOA, but TOA requires clock synchronization between each Anchor and Tag while the TDOA model only requires clock synchronization between the Anchors. TDOA hardware can be more simply and easily implemented, to this effect, than TOA hardware. According to the hyperbolic characteristics, Eq. (3) can be obtained:

3 $$\left\{ \begin{array}{ll} \sqrt{(x_2-x_0)^2+(y_2-y_0)^2}-\sqrt{(x_1-x_0)^2+(y_1-y_0)^2}&=r_{21}\\ \sqrt{(x_3-x_0)^2+(y_3-y_0)^2}-\sqrt{(x_1-x_0)^2+(y_1-y_0)^2}&=r_{31}\\ &\cdots \\ \sqrt{(x_n-x_0)^2+(y_n-y_0)^2}-\sqrt{(x_1-x_0)^2+(y_1-y_0)^2}&=r_{n1}\\ \end{array} \right.$$

The Anchors coordinates are known, $(x_i,y_i)$ represents the *i*th Anchors location, and $(x_0,y_0)$ represents the Tags coordinate which which can be calculated via Eq. (3).

#### Smoothing method based on Kalman filter for TDOA measurements

The Kalman smoothing algorithm is applicable to TOA and TDOA distance measurements. The measurements should meet two conditions: (1) A previous estimated value $P_{k-1}$ and (2) The time interval *T* between current point and last point less than the threshold $\lambda$. When these conditions are met, $P_{k-1}$ can be used as Kalman filter parameters to obtain the estimated value $P_k$. Otherwise, measurements are the necessary parameters to determine $P_k$ directly. As discussed in detail below, some results are disturbed severely by NLOS. When this occurs, the state estimated values of $P_k$ (assuming at time $t_k$) will be given up and previous estimated values of $P_{k-1}$ will be reused in the subsequent iteration. Because the Kalman filter as a linear optimal filtering algorithm can effectively utilize historical data, it also smooths the measurements and effectively reduces error. The following pseudo-code describes the Kalman-based smoothing process:

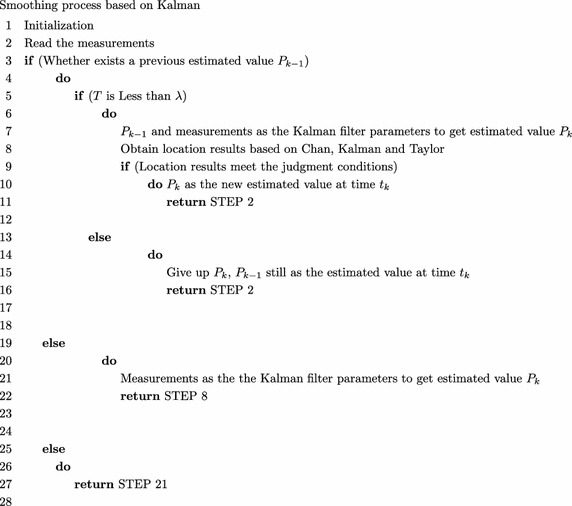



#### Cooperative localization based on Chan, Kalman, and Taylor

Chan TDOA algorithms can be utilized to linearize TDOA hyperbolic equations and conduct dual weighted least squares (WLS) to obtain useful results; this yields relatively high accuracy if the noise error is in Gaussian distribution. Taylor TDOA algorithms are recursive, and require an initial estimated value to expand the Taylor series and linearize nonlinear equations, so the calculation load is high (and the results divergent) when there are large initial errors. The EKF lends the Kalman-based TDOA algorithm better dynamic performance. Based on the features of the three different algorithms, we developed a cooperative localization method based on Chan, Taylor and Kalman; three algorithms are used to calculate the location results while setting thresholds for the residual sum of the squares to identify the NLOS error.

Per the proposed method, the measured data (smoothed by a Kalman filter) is read first and initial results $(x_1, y_1)$ are obtained via Chan algorithm. The residual sum of squares of the Chan algorithm $(Res_{chan})$ are then compared with the first threshold $\delta _1$. The residual square sum of estimation result (*x*, *y*) is defined as follows:

4 $$ R_{es}=\sum _{i=2}^n\left(\sqrt{(x_i-x)^2+(y_i-y)^2} -\sqrt{(x_1-x)^2+(y_1-y)^2}-r_{i1}\right)^2 $$

where *n* is the number of Anchors, $(x_i, y_i)$ is the coordinate of the *i*th Anchor, $r_{i1}$ is the difference in distance between the *i*th Anchor and the first Anchor (i.e., reference Anchor).

If the first result of the Chan algorithm is less than the first threshold, it serves as the initial value of the Taylor algorithm to obtain a second result $(x_2, y_2)$. Then, the second threshold $\delta _2$ is set for judging the $Res_{taylor}$ (residual sum of squares) of $(x_2, y_2)$, as mentioned above.

Third, there are two necessary conditions remaining for the $Res_{taylor}$: Whether the Kalman algorithm has been initialized, and whether the interval time is below threshold $\delta _5$. If both are satisfied, $(x_2, y_2)$ will be the input parameter of the Kalman algorithm to obtain another result $(x_3, y_3)$, or the estimated results $(x_2, y_2)$ will be the initial value of the Kalman algorithm and become the final result. Then, provided there is sufficiently small measurement error, the location results of Taylor and Kalman are close to each other based on these characteristics. (The larger the error, the larger the deviation). Another two thresholds, $\delta _3$ and $\delta _4$, are adopted to judge two types of inequality and further determine whether the measurement error is too large (Eq. 5). If both threshold conditions are not met, the process returns to the first step to read the measurement and gives up the current step.

5 $$\begin{aligned} |x_3-x_2|+|y_3-y_2|<\delta _3\quad or \quad |x_2-x_{k-1}|+|y_2-y_{k-1}|<\delta _4 \end{aligned}$$

where $(x_{k-2}, y_{k-1})$ is the ultimate estimated result at the time $t_{k-1}$. Again, when the estimated results $(x_2, y_2)$ and $(x_3, y_3)$ do not meet the condition of Eq. (5), the next step is given up and the measured value must be re-read. Otherwise, the residual square sum $Res_{kalman}$ of the Kalman algorithm is calculated and the residual weighted method is applied to obtain location result $(x_4, y_4)$ Eq. (6).

6 $$\begin{aligned} \begin{aligned} x_4&=\frac{{Res_{kalman}}*{x_3}+{Res_{taylor}}*{x_2}}{Res_{kalman}+Res_{taylor}}\\ y_4&=\frac{{Res_{kalman}}*{y_3}+{Res_{taylor}}*{y_2}}{Res_{kalman}+Res_{taylor}}\\ \end{aligned} \end{aligned}$$

In summary, the Chan, Taylor, and Kalman algorithms are used in sequence across the whole positioning computational procedure as illustrated in the following pseudo-code.

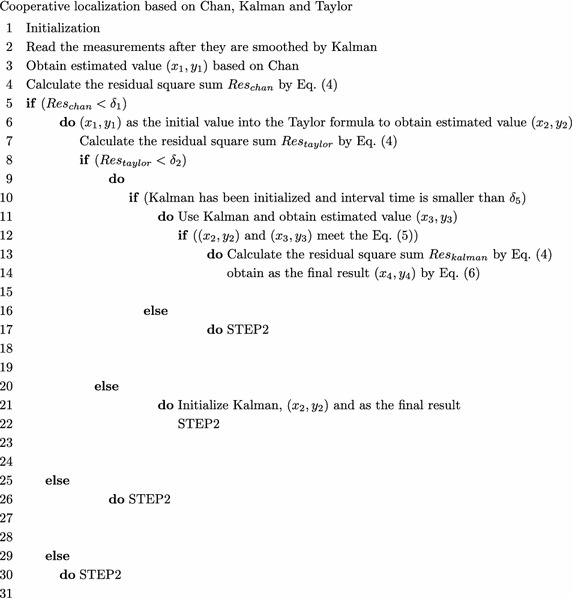



The values of the thresholds $\delta _1, \delta _2, \delta _3$ and $\delta _4$ are highly influenced by the accuracy of the historical data and the precision of the location device; when they are appropriate, they reduce the interference of NLOS and improve the location accuracy overall. We conducted several experiments yielding a large amount of measured data which were used to get location results by the three algorithms (Chan, Taylor, and Kalman). $\delta _1$ and $\delta _2$ first determined by the residual sum of squares of the location results of Chan and Taylor separately. $\delta _3$ and $\delta _4$ first determined by the difference value between the mean value of the residual sum of squares of the location results of Taylor and Kalman. Then adjusted the threshold values according to the actual effect. Finally, the thresholds were set by the following principles iteratively and verified according to the accuracy of the location results. (1) $\delta _1$ is set as large as possible to filter the measurements with large error and reduce the calculation amount of the Taylor algorithm. (2) $\delta _2$ is set carefully to discard the measurements which suffer from excessive NLOS. Otherwise, the accuracy of the whole method would be severely affected. (3) $\delta _3$ and $\delta _4$ validate whether the measurements meet the necessary criteria, then measurements suffering excessive NLOS are further discarded.

#### Smoothing method based on moving weighted average for location results

The moving weighted average method can be used to smooth the location result $(x_4, y_4)$ obtained in the previous step at time $t_k$. An abundance of historical data is necessary to smooth the result; the thresholds $\delta _6, \delta _7$ restrict the time and space of said data, respectively, as expressed in Eqs. (7) and (8). The history data $(x_i, y_i)$ at time $t_i$ must satisfy both.

7 $$\begin{aligned} |t_k-t_i|<\delta _6 \end{aligned}$$

8 $$\begin{aligned} \sqrt{(x_4-x_i)^2+(y_4-y_i)^2}<\delta _7 \end{aligned}$$

After securing qualified data, each weighting coefficient is given and the last estimated results $(\hat{x}, \hat{y})$ are obtained via Eq. (9):

9 $$\begin{aligned} \begin{aligned} \hat{x}&=\frac{\sum _{i=1}^{m}q_ix_i+qx_4}{\sum _{i=1}^{m}q_i+q}\\ \hat{y}&=\frac{\sum _{i=1}^{m}q_iy_i+qy_4}{\sum _{i=1}^{m}q_i+q}\\ \end{aligned} \end{aligned}$$

where $(x_i, y_i)$ is the selected historical location result, $q_i$ is the corresponding weighting coefficient, $(x_4, y_4)$ is the current estimation result,and *q* is the corresponding weighting coefficient. The smaller the *i* value, the closer the historical result $(x_i, y_i)$ is to the current estimated result $(x_4, y_4)$ in time and space. This result is then referenced to assign the value of weighting coefficients, which we set to $q=0.5, q_1=0.25, q_2=0.125, q_3=0.0625, q_i=2^{-(i+1)}$.

### Design and implement of location management server

The location management server is responsible for storing data and managing communications with the Location Engine and Anchor devices. To facilitate server management operations, the location server was divided into two parts according to the pre-established software framework design: Data Server and MClient. Data Server is an information processing center and Mclient is an information management center.

#### Data server implementation

The Data Server integrates both TCP and UDP network protocol and has two server-sides plus a data processing module. The server programs use C++ SOCKET network programming and an event selection (WSAEventSelect) asynchronous I/O model. An information processing module phases the received request data, and a server sends the corresponding response data. The structure of this framework is illustrated in Fig. 4.

As shown in Fig. 4, the data server is defined by two-layers to facilitate management. The bottom layer or so-called Data Communication Layer is mainly tasked with data communication. The top layer, the Data Application Layer, generates response data through a data analysis process which is later transmitted to the Data Communication Layer and sent to the appropriate client according to the response rule. Consequently, the connected objects and appropriate clients can be divided into three categories: (1) MClient (Interface Management Client), (2) LEClient (Location Engine Client), and (3) RawDataClient (Providing Measurement Client). Among them, RawDataClient uses UDP as a communication protocol, LEClient uses TCP, and MClient uses both.

All previously referred data are received by the Communication Layer and processed by the Application Layer. The response data must contain a TCP address and be cached in the Communication Layer. Finally, the location results are received and displayed by MClient; the received data are stored in the database. A flow chart of this process is shown in Fig. 5.

#### MClient implementation

Mclient is the management center for resource information in the proposed system; it performs data synchronization with the Data Server. MClient interacts with the database and can display location results graphically in real-time. With the interface, administrators can set configuration parameters for each module and manage the system resources efficiently and intuitively. MClient is divided into the following function modules dependent on the type of data: (1) The location information display module, which displays location results in real-time in graphic form. (2) The network address information module, which sets the IP and port of MClient. (3) The anchor information module, which sets Anchor information (such as Anchor additions, coordinate information, and measurement error). (4) The Tag information module, which operates and monitors Tag information. (5) The location zone information module, which manages zone information (such as map information and work state). (6) The algorithm parameter information module, which sets the algorithm parameters.

Initiating a positioning area to a start position requires two factors: First, that all Anchors in the zone have run and been registered in Data Server; and second, that at least one Location Engine has run. When these requirements are met, one Location Engine is selected as the computing center. A command message containing relevant Anchor information and selected Location Engine information is then sent to the Data server. The Data server detects whether the Anchors and Location Engine are ready; once ready, the Data server runs the corresponding program and readies for location. Location results can be obtained from the Location Engine and sent to the Data Server at the same time. Figures 6 and 7 show images of the MClient interface.

## Results

### Wireless location test design

The Nanotron nanoLOC Development Kit 3.0 suite (Nanotron Technology) was used to simulate a small zone based on its Location Demo. We built a simulated location system and ran a location test with the Nanotron Location Server and four other parts (the RawDataClient, Data Server, Mclient, and LEClient). We first set up the Location Server and placed the Tag in a fixed position, then ran all programs and set all system configuration parameters according to the physical parameters. After powering on the Anchors and Tag and clicking the start button, MClient sent the ranging command to force the Anchors and Tag to start ranging. Next, each program module of the location system ran concurrently while location results were calculated and displayed in real-time as shown in Fig. 7.

We designed both indoor and outdoor location experiments to evaluate the performance of this location system. We compared the location accuracy of five algorithms altogether including Chan, Taylor (where Chan provides the initial estimated value), Kalman, Compared Taylor Method (where a threshold is set for residual square summation to select the result), and cooperative localization method based on Kalman and Taylor (where location results were smoothed). We also evaluated the smoothing algorithm based on Kalman for ranging data in regards to location accuracy. Positioning accuracy, i.e., the error between the estimated position and real position, is the most import criterion for a positioning algorithm. We adopted the root mean squared error (RMSE) and absolute error to evaluate the various positioning methods.

### Indoor location test

Four Anchors, Anchor 1 (0.00, 0.00), Anchor 2 (5.65, 0.00), Anchor 3 (5.65, 5.40), Anchor 4 (0.00, 5.40), were placed in a laboratory at Hangzhou Dianzi University 8.3 m × 8.5 m as a small zone within which a Tag was placed in a certain position. This area represents a complex environment through which people move frequently in and out among work tables, computers, and other equipment.

Through statistical analysis of experimental data, the following parameters were set: $\lambda =5000\,{\mathrm{ms}}, \delta _{1} =4, \delta _{2}=0.36, \delta _3=0.14, \delta _4=0.5, \delta _5=5000\,{\mathrm{ms}}, \delta _6=2000\,{\mathrm{ms}}, \delta _7=1.5\,{\mathrm{m}}$; each was based on the measured data and the actual effects of the experiment. $\lambda$ was used to smooth the measurements based on the Kalman algorithm to limit the time interval; $\delta _1, \delta _2, \delta _3, \delta _4$ and $\delta _5$ were applied to complete the cooperative localization method based on Chan, Kalman, and Taylor; $\delta _6$ and $\delta _7$ were applied to smooth the results. These parameters were repeatedly set during the experiment until finally being established as discussed above to secure the optimal results. In practice, only TOA ranging data were collected, so an Anchor was set as the reference and TDOA data was obtained via algorithm measurements (Additional file 1).

Tag was placed in six different positions and six sets of ranging data were gathered. Chan, Taylor, Kalman, Compared Taylor Method, and method in this paper were respectively used. Then, location results and corresponding RMSE were given as Tables 1 and 2.

### Outdoor location test

Four Anchors, Anchor 1 (0.00, 0.00), Anchor 2 (5.00, 0.00), Anchor 3 (5.00, 8.00), Anchor 4 (0.00, 8.00), were placed on a university campus lawn as a small zone and a Tag was placed at a certain position within the zone. The following parameters were set: $\lambda =5000\,{\mathrm{ms}}, \delta _ 1 = 4, \delta _2=0.36, \delta _3=0.14, \delta _4=0.5, \delta _5=5000\,{\mathrm{ms}}, \delta _6=2000\,{\mathrm{ms}}, \delta _7=1.5\,{\mathrm{m}}$.

The Tag was placed in three different positions to gather three sets of ranging data, then Chan, Taylor, Kalman, Compared Taylor Method, and Cooperative localization Method Based on Kalman and Taylor were respectively used for comparison. The location results and corresponding RMSE values are provided in Tables 3 and 4.

### Comparative experiments

At first, the partial ranging data of the indoor position (3.39, 5.40) and outdoor position (3.00, 4.00) were respectively used to compare the location errors of Taylor and Kalman methods, as illustrated in Figs. 8 and 10. Location errors among the Taylor and Kalman/Taylor methods were also compared as illustrated in Figs. 9 and 11.

To verify whether the Kalman-based smoothing algorithm for ranging data improved the location accuracy and enhanced robustness, partial ranging data of the indoor position (3.39, 5.40) and outdoor position (3.00, 4.00) were investigated: The ranging data were divided into two test groups (those smoothed via Kalman method before being calculated by cooperative localization, and those obtained directly via cooperative localization) while two other groups of location results were smoothed by moving weighted average method. The absolute location error among the groups was compared as shown in Figs. 12 and 13.

## Discussion

Tables 1, 2, 3, and 4 clearly show that Chan was less accurate than Taylor/Kalman or the proposed method. Taylor, Kalman, and Compared Taylor had almost the same RMSE, while Taylor/Kalman had smaller RMSE. The RMSE value reflects the bias between estimated and real results; the smaller the RMSE, the greater the accuracy. The experimental cooperative localization results of the cooperative Kalman and Taylor method was highly accurate. The indoor results were worse than the outdoor results due to NLOS. The RMSE of the proposed method was the lowest out of all the location methods, at below 0.6.

Figures 8 and 9 show where the cooperative method resulted in absolute error lower than 0.7 m and average error of about 0.5 m, while the absolute errors of Taylor, Kalman, and compared Taylor were close to 1 m in the indoor test. Figures 10 and 11 show where the cooperative method performed better than the other three methods in the outdoor location test as well, with absolute errors smaller than those of the indoor test (slightly below 0.6 m).

As shown Figs. 12 and 13, the Kalman-based smoothing method indeed yielded more stable location results, especially in the outdoor experiment. Furthermore, measured values were smoother and ranging error was smaller after applying the smoothing method. The cooperative localization method not only improved the efficiency, but also further restrained NLOS, yielding higher accuracy overall.

## Conclusions

A location system based on the CSS signal and TDOA method was developed in this study. The proposed system is comprised of a Location Engine and a Location Server. To verify its feasibility and effectiveness, we conducted indoor and outdoor experiments in real-time using a NanoLOC Development Kit 3.0. The proposed localization method does not have favorable adaptability, as the parameters $\delta _1, \delta _2, \delta _3, \delta _4$ and $\delta _5$ are not self-adaptive; when the environment changes, these parameters should change as well. However, when these parameters are appropriate for the environment (such as in the test we conducted), the proposed cooperative localization method based on Kalman and Taylor did restrain NLOS effectively while the Kalman-based smoothing algorithm reduced the measurement error and enhanced the robustness of the system on the whole.

The next step in developing the proposed method is to improve the parameter selection process to make it better adaptable to different situations. The system program was able to run stably with high accuracy, so the work done in this study is meaningful, but to further improve the proposed systems accuracy, it remains necessary to (1) build localization sensor nodes with higher precision (including Anchors and Tags) and (2) to fully optimize the localization algorithm.

## Additional file

**Additional file 1.** Experimental Data. This file includes experimental row data collected during the experiments, and the format of data files is txt file. (RAR 811 kb)

## Abbreviations

LBS: location based services
WSN: wireless sensor networks
CSS: chirp spread spectrum
TDOA: time difference of arrival
GPS: global positioning systems
UWB: ultra-wide bands
NLOS: non-line of sight
RFID: radio frequency identification
AOA: angle of arrival
RSSI: received signal strength indication
TOA: time of arrival
EKF: extended Kalman filter
MClient: management client
WLS: weighted least squares
